# Knowledge, Awareness and Practice of Artificial Intelligence and Types of Realities Among Healthcare Professionals: A Nationwide Survey From Pakistan

**DOI:** 10.7759/cureus.57695

**Published:** 2024-04-05

**Authors:** Haseeb Mehmood Qadri, Momin Bashir, Manal Khan, Arham Amir, Allah Yar Yahya Khan, Zainab Safdar, Hassan Chaudhry, Usama Afraz Younas, Asif Bashir

**Affiliations:** 1 Neurological Surgery, Punjab Institute of Neurosciences, Lahore, PAK; 2 Microbiology, New York University, New York, USA; 3 General Surgery and Surgical Oncology, Shaikh Zayed Medical Complex, Lahore, PAK; 4 Surgery, Farooq Hospital, Lahore, PAK; 5 General Surgery, Lahore General Hospital, Lahore, PAK; 6 Surgery, Rashid Latif Medical College, Lahore, PAK; 7 Surgery, Jinnah Hospital Lahore, Lahore, PAK

**Keywords:** augmented reality, virtual reality, healthcare tech contest, awareness, knowledge, pakistan, healthcare technology, healthcare tech, technology

## Abstract

Background

Artificial intelligence (AI) refers to the simulation of human intelligence processes by machines, enabling them to perform tasks. The advancements in AI have also improved virtual reality (VR), augmented reality (AR) and mixed reality (MR) experience allowing a greater opportunity for use in the field of medicine.

Objective

To evaluate the knowledge, attitude and practice of AI and types of realities among Pakistani healthcare professionals (HCPs).

Materials and methods

This was a prospective, nationwide study designed at the Department of Neurosurgery at Punjab Institute of Neurosciences (PINS), Lahore, was conducted between January 2024 to February 2024. More than 500 HCPs were approached, out of which 176 participated in this survey consensually. A pre-formed general questionnaire based on knowledge, attitude and practices of AI and types of realities was modified according to local conditions. Google Forms (Google Inc., USA) was used to conduct the one-time sign up response. Statistical Package for Social Sciences (IBM SPSS Statistics for Windows, Version 24, USA) was used to analyze submitted responses.

Results

About 69.9% respondents were male HCPs. Most of the respondents were from the fields of neurosurgery, medicine and general surgery, i.e., 10.80%, 10.20% and 4%, respectively. More than 90% HCPs used Internet and electronic devices daily. A majority of 62.50% respondents agreed that AI brings benefits for the patients, while at the same time, 45.50% agreed that they would not trust the assessment of AI more than that of HCPs. 61% HCPs feared that AI-based systems could be manipulated from the outside sources, like terrorists and hackers. Although 90% respondents knew the definition of AR and VR, a strikingly low 40% respondents could only identify the practical applications of these realities when asked in a mini-quiz. About 61.40% HCPs never used any AI-based application throughout their clinical practice, but Google Health was used by 29.50% respondents, followed by Remote Patient Monitoring AI application used by 3.4% individuals.

Conclusion

There is an evident under-utilization of AI and types of realities in clinical practice in Pakistan. Lack of awareness, paucity of resources and conventional clinical practices are the key reasons identified. Pakistan is on the path towards the point where the developed world is currently. There is a potential to move past the initial stages of AI implementation and into more advanced modes of adopting AI and types of realities.

## Introduction

Artificial intelligence (AI) has emerged as a transformative force, reshaping various aspects of human life. Although the task of defining Al can be cumbersome, a definition given by Darthmouth Research Project in 1955 still holds relevance: "making machine behave in ways that would be called intelligent if a human were so behaving" [1]. In other words, Al refers to the simulation of human intelligence processes by machines, enabling them to perform tasks that typically require human intelligence. Primarily, there are three types of AI. Narrow Al is a specialized Al designed to perform a specific task, which includes Siri or Alexa. General AI possesses human-like cognitive ability and can learn, understand, and apply its knowledge across various domains. Artificial super-intelligence, a theoretical Al that surpasses human intelligence across all domains [1].

The advancements in AI have also improved virtual reality (VR), augmented reality (AR) and mixed reality (MR) experience allowing enhanced realism, object recognition, tracking and natural language processing, thus allowing a greater opportunity for use in the field of medicine. VR refers to a simulated experience that involves the use of technology to create a simulated environment that users can interact with and immerse themselves in through various sensory stimuli, like sight, sound and touch. This simulated environment can be similar to or completely different from the real world [2]. AR essentially augments the real-world environment by adding virtual elements to it while utilizing devices like smartphones, tablets and special glasses that add digital content to a person’s field of view in real time. AR uses real world settings while VR is completely virtual. MR, as the name implies, is a hybrid environment that merges aspects of both AR and VR. This allows coexistence of virtual and real-world elements and enables interaction between the digital and real world [1,2].

AI allows complex pattern recognition in a multidimensional data set making it possible for it to analyze different images in the fields of radiology, pathology, dermatology, and ophthalmology to delineate various lesions and pathologies [3]. The processing of a large amount of data in lesser time would help supplement the limited processing power of a human mind and help to reduce health care professional workload. VR and AR also hold promise in physician training allowing surgical trainees to practice on virtual patients that would vastly reduce the possibility of surgical errors and subsequently improve patient safety [1,2]. Although the potential of AI holds promises in the field of medicine, some potential demerits should also be kept in mind. AI automation can cause job displacement when tasks that are traditionally performed by humans get replaced by AI. This could potentially lead to unemployment and socio-economic disruption. Decisions by AI can be biased based on the data its developed on, leading to unfair or discriminatory outcomes. Not to mention the distrust of the public towards AI could potentially pose challenges in integrating it in a health care setting [1].

Despite the rapid growth of research on AI in healthcare globally, there is a noticeable lack of studies on this topic in Pakistan. While some researches have explored perspectives of medical students and post graduates, there is a significant lack in the formal studies targeting medical consultants and healthcare professionals (HCPs) [1,3]. We aimed to conduct a comprehensive study focusing on HCPs in Pakistan to achieve their insight, experiences, and concerns regarding the use of AI in healthcare practice. 

## Materials and methods

This was a prospective cross-sectional study conducted by the Department of Neurosurgery, Unit-I, Punjab Institute of Neurosciences (PINS), Lahore. The study was conducted in January and February 2024. It was a nationwide survey which targeted HCPs. Non-probability, convenience sampling was used as a sampling technique.

HCPs who completed their specialization degrees and were currently practicing in Pakistan were targeted in this survey. These included professionals who were awarded specialization degrees namely, Fellow of College of Physicians and Surgeons (FCPS), Master of Surgery (MS), Doctor of Medicine (MD), Master in Dental Surgery (MDS), Member of College of Physicians and Surgeons (MCPS), Master of Philosophy (MPhil) and professionals with international equivalent medical qualifications. There was no restriction on the field of human medicine to which the HCPs belonged. Pakistani HCPs belonging to the fields of basic sciences namely, anatomy, physiology, biochemistry, pharmacology, forensic sciences, community medicine and those practicing outside Pakistan were not part of the study.

A pre-formed general questionnaire in English language based on knowledge, attitude and practices of AI and types of realities was modified according to local conditions [1]. The survey questionnaire had six sections, the preliminary section detailing the nature and purpose of the questionnaire and an oath as mentioned-below. The sections A, B, C, D and E had multiple questions targeting demographic and work details, evaluation of respondent's technical affinity, attitudes and practice of different aspects of AI in healthcare, knowledge of types of realities and survey feedback, respectively.

Google Forms (Google Inc., USA) was used to conduct the one-time sign up response. Authors were allowed to contact as many HCPs as possible according to the set criteria throughout Pakistan. Utilization of social media and close contacts was encouraged to disseminate the form. There was an oath included in the questionnaire survey mentioning the anonymity of the survey responses and ensuring a pledge that the HCPs would consent to respond as per their interest, will and honesty. More than 500 HCPs were approached by the ambassadors but 176 responded. Statistical Package for Social Sciences (IBM SPSS Statistics for Windows, Version 24, USA) was used to analyze the submitted responses in terms of measures of central tendency, percentage and frequency. 

The Departmental Review Board, Neurosurgery-I of PINS, Lahore, issued an exemption letter for this web-based survey study, reference# 49/NS-I/2024, dated March 10, 2024.

## Results

**Figure 1:**
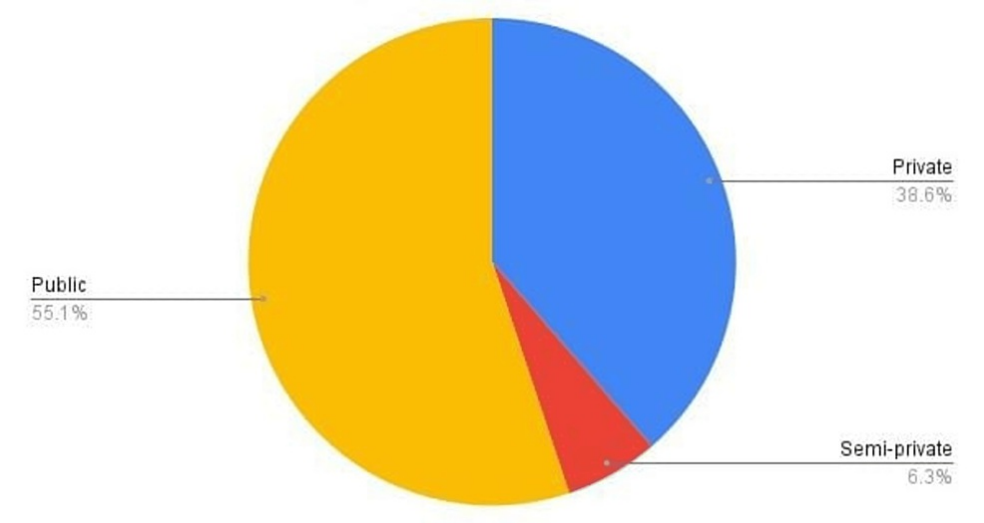
Type of healthcare hospital of included respondents in terms of percentage, %.

Out of 176 responses 69.90% were male HCPs. The mean age of respondents was 41.86 ± 11.24 years. About 55.10% of the responses were from HCPs working at public sector hospitals (Figure 1).

**Figure 2:**
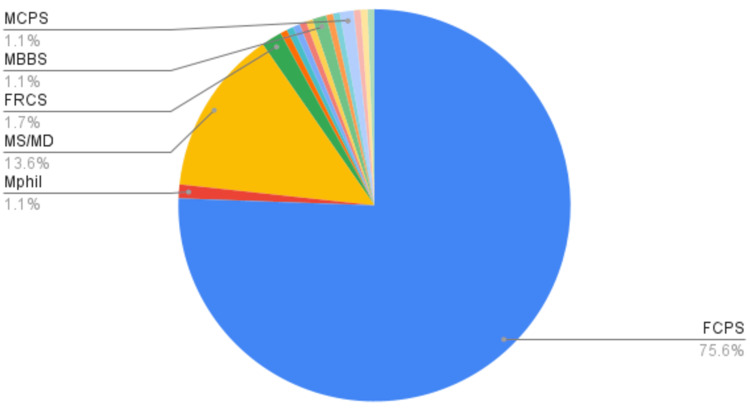
Type of degrees received by included HCPs, in terms of percentage, %. HCP: Healthcare professional

**Figure 3:**
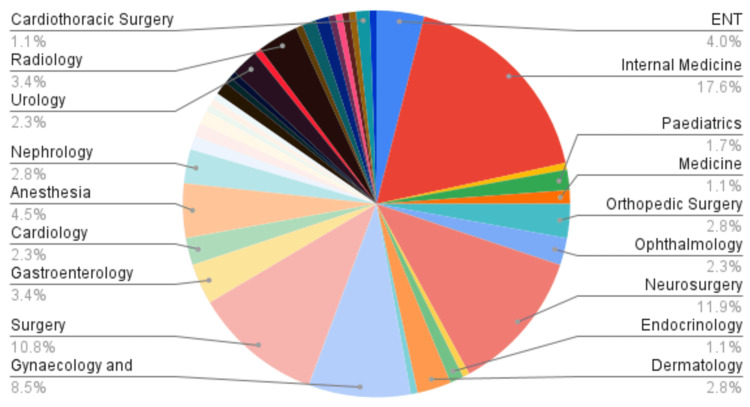
Field specialties of included HCPs in terms of percentage, %. HCP: Healthcare professional

Among the degree programs, most of the HCPs had acquired the Pakistani degree, namely FCPS. Most of the specialist consultants were from the fields of Internal Medicine, Neurosurgery, and Surgery, i.e., 17.60%, 11.90%, and 10.80%, respectively (Figures 2, 3).

A whopping majority of 95.50% HCPs used the Internet once daily and 97.70% used it via their smartphones or tablets (Tables 1, 2).

**Table 1:** Frequency, n, and percentage, %, of respondents using the Internet and electronic devices.

Sr. #	Questions	Daily % (n)	More than three times per week % (n)	Less than three times per week % (n)	More than one time per month % (n)	Never % (n)
1.	How often do you use the Internet?	95.50% (168)	2.30% (4)	1.10% (2)	0.60% (1)	0.60% (1)
2.	How often do you use a computer or similar electronic device (tablet, smartphone, etc.)?	90.90% (160)	4.50% (8)	2.30% (4)	1.70% (3)	0.60% (1)

**Table 2:** Frequency, n, and percentage, %, of respondents using gadgets.

Sr #.	Questions	Yes % (n)	No % (n)
1.	Do you use a smartphone or tablet?	97.70% (172)	2.30% (4)
2.	Do you use medical apps or computer programs?	90.90% (160)	9.10% (16)

More than 50% HCPs found it easy to get accustomed to a new technology, and more than 70% felt confident of using technology gadgets (Tables 3, 4).

**Table 3:** Level of difficulty observed by respondents when using a new device, function or program, in terms of percentage, %, and frequency, n.

Questions	Very Difficult % (n)	Difficult % (n)	Neutral % (n)	Easy % (n)	Very Easy % (n)
How difficult is it for you to familiarize yourself with a new device, a new program, or a new function of a device?	1.70% (3)	17.60% (31)	30.10% (53)	42.60% (75)	8.00% (14)

**Table 4:** Confidence of respondents in using computer and other electronic devices, in terms of percentage, %, and frequency, n.

Questions	Very unconfident % (n)	Unconfident % (n)	Neutral % (n)	Confident % (n)	Very confident % (n)
How confident (skilled) do you feel in using computers and other electronic devices (smartphone, tablet, etc.)?	1.70% (3)	5.10% (9)	21.00% (37)	52.80% (93)	19.30% (34)

Although Pakistani HCPs had an overall positive attitude towards AI and types of realities, but their practice was almost none. There were mixed responses (Table 5, 6).

**Table 5:** Attitude and practice of different aspects of AI in healthcare by HCPs, in terms of percentage, %, and frequency, n. AI: Artificial intelligence; HCP: Healthcare professional

Sr. #	Questions	Strongly Disagree % (n)	Disagree % (n)	Neutral % (n)	Agree % (n)	Strongly Agree % (n)
1.	I think that the use of AI brings benefits for the patients.	0% (0)	5.70% (10)	18.80% (33)	62.50% (110)	13.10% (23)
2.	Physicians will play a less important role in the therapy of patients in the future.	15.90% (28)	51.10% (90)	14.80% (26)	15.90% (28)	2.30% (4)
3.	With AI, there will be less treatment errors in the future.	2.30% (4)	26.70% (47)	30.10% (53)	35.80% (63)	5.10% (9)
4.	AI should not be used in medicine as a matter of principle.	6.80% (12)	33.50% (59)	18.70% (33)	35.80% (63)	5.10% (9)
5.	Physicians are becoming too dependent on computer systems.	2.80% (5)	19.30% (34)	25.00% (44)	46.60% (82)	6.30% (11)
6.	The testing of AI before it is used on patients should be carried out by an independent body.	1.10% (2)	1.70% (3)	8.00% (14)	57.40% (101)	31.80% (56)
7.	I would trust the assessment of AI less than my assessment as a healthcare professional.	8.50% (15)	18.80% (33)	15.90% (28)	45.50% (80)	11.40% (20)
8.	Physicians know too little about AI to use it on patients.	0.6% (1)	6.20% (11)	14.80% (26)	65.90% (116)	12.50% (22)
9.	If a patient has been harmed, a physician should be held responsible for not following the recommendations of AI.	13.10% (23)	50.00% (88)	18.20% (32)	16.50% (29)	2.30% (4)
10.	The influence of AI on medical treatment scares me.	3.40% (6)	27.80% (49)	24.40% (43)	39.20% (69)	5.10% (9)
11.	The use of AI prevents physicians from learning to make their own correct judgement of the patient.	4.50% (8)	15.90% (28)	17.60% (31)	53.40% (94)	8.50% (15)
12.	If AI predicts a low chance of survival for the patient, physicians will not fight for that patient’s life as much as before.	10.80% (19)	31.80% (56)	15.90% (28)	39.20% (69)	2.30% (4)
13.	The use of AI is changing the demands of the medical profession.	1.70% (3)	5.10% (9)	14.20% (25)	70.50% (124)	8.50% (15)
14.	I would make my anonymous patient data available for non-commercial research (universities, hospitals, etc.) if this could improve future patient care.	2.30% (4)	6.80% (12)	9.70% (17)	70.50% (124)	10.80% (19)
15.	AI-based decision support systems for physicians should only be used for patient care if their benefit has been scientifically proven.	0.6% (1)	1.10% (2)	8/00% (14)	66.50% (117)	23.90% (42)
16.	I am more afraid of a technical malfunction of AI than of a wrong decision by a physician.	1.10% (2)	10.80% (19)	20.50% (36)	54/00% (95)	13.60% (24)
17.	I am not worried about the security of patient data.	11.90% (21)	43.80% (77)	18.20% (32)	23.90% (42)	2.30% (4)
18.	A physician should always have the final control over diagnosis and therapy.	1.70% (3)	0.60% (1)	2.80% (5)	40.30% (71)	54.50% (96)
19.	I am worried that AI-based systems could be manipulated from the outside (terrorists, hackers, etc.).	0% (0)	5.10% (9)	15.30% (27)	61.40% (108)	18.20% (32)
20.	The use of AI impairs the physician-patient relationship.	0.60% (1)	14.20% (25)	15.90% (28)	58.00% (102)	11.40% (20)
21.	The use of AI is an effective instrument against the overload of physicians and the shortage of physicians.	2.80% (5)	14.80% (26)	30.10% (53)	48.90% (86)	3.40% (6)
22.	I would override the recommendations of AI if I come to a different conclusion from AI.	0.60% (1)	5.10% (9)	17.60% (31)	59.70% (105)	17.00% (30)
23.	The use of AI will reduce the workload of physicians.	1.10% (2)	12.50% (22)	24.40% (43)	55.10% (97)	6.80% (12)

**Table 6:** Overall impression of respondents towards AI, in terms of percentage, %, and frequency, n. AI: Artificial intelligence

Question	Very Negative % (n)	Negative % (n)	Neutral % (n)	Positive % (n)	Very Positive % (n)
Taken all together: How positive or negative do you feel about the use of AI in medicine?	1.70% (3)	11.40% (20)	25.00% (44)	56.30% (99)	5.70% (10)

**Figure 4:**
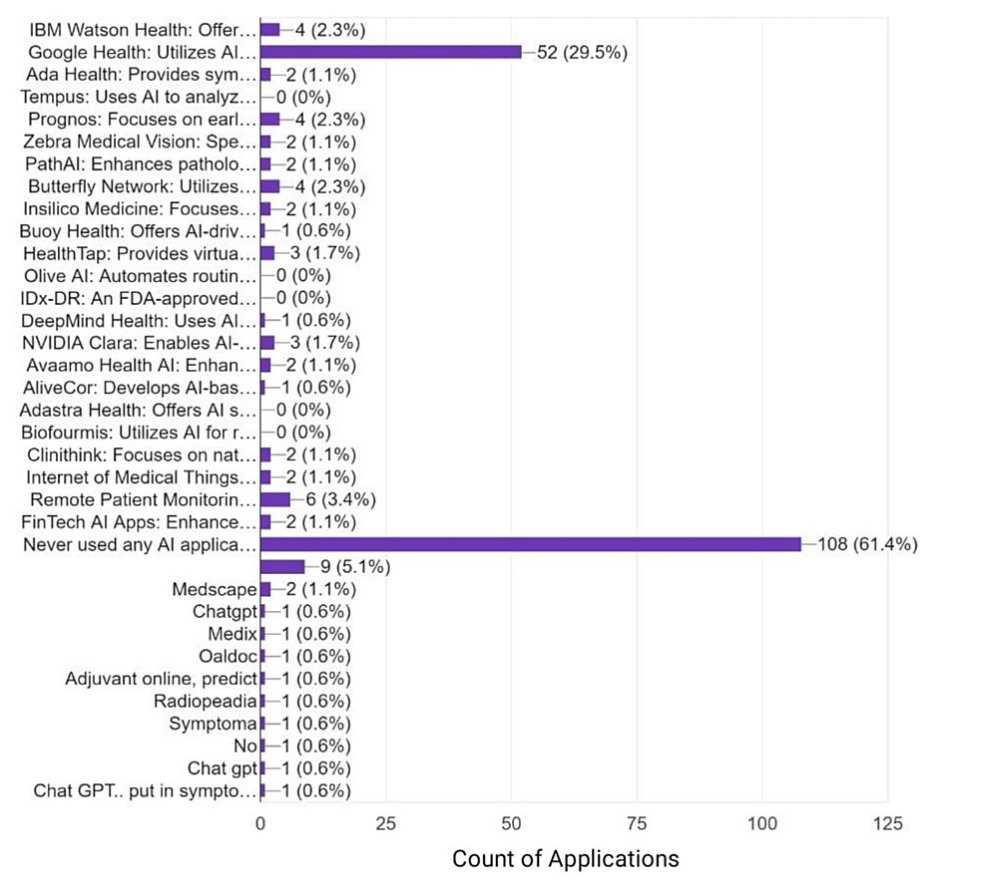
AI applications used by Pakistani HCPs, in terms of percentage, %. IBM Watson Health: Offers advanced data analytics for personalized treatment plans. Google Health: Utilizes AI for medical imaging and health data analysis. Ada Health: Provides symptom-checking and personalized health insights. Tempus: Uses AI to analyze clinical and molecular data for cancer care. Prognos: Focuses on early disease detection through AI-driven analytics. Zebra Medical Vision: Specializes in radiology AI for imaging analysis. PathAI: Enhances pathology diagnostics using machine learning. Butterfly Network: Utilizes handheld ultrasound devices with AI for diagnostics. Insilico Medicine: Focuses on drug discovery and aging research. Buoy Health: Offers AI-driven virtual triage and symptom checking. HealthTap: Provides virtual care and medical advice through AI. Olive AI: Automates routine administrative tasks in healthcare settings. DeepMind Health: Uses AI for various healthcare applications, including patient monitoring. NVIDIA Clara: Enables AI-powered medical imaging and genomics. Avaamo Health AI: Enhances patient engagement and care coordination. AliveCor: Develops AI-based tools for heart monitoring and ECG analysis. Adastra Health: Offers AI solutions for clinical trial optimization. Biofourmis: Utilizes AI for remote patient monitoring and predictive analytics. Clinithink: Focuses on natural language processing. IoMT: Enables connectivity and data exchange among medical devices, improving real-time monitoring and remote patient management. RPM AI Apps: Facilitate continuous monitoring of patients' health outside traditional healthcare settings. FinTech AI Apps: Enhance financial services by automating processes, such as improving fraud detection. Never used any AI application for healthcare purposes. AI: Artificial intelligence; HCP: Healthcare professional; IoMT: Internet of medical things; RPM: Remote patient monitoring

Google Health was the most commonly used application used by Pakistani HCPs, while more than two-thirds of HCPs never used any AI-related application (Figure 4).

More than 90% HCPs knew the definitions of types of realities, but paradoxically approximately 40% knew the correct answers to the practical applications of AR, VR and MR (Tables 7, 8).

**Table 7:** Knowledge of definitions of types of realities, in terms of percentage, %, and frequency, n. Correct answers are formatted in bold text. AR: Augmented reality; VR: Virtual reality

Sr. #	Statements	True % (n)	False % (n)
1.	AR does not replace the real world but adds computer-generated elements, such as images, sounds, or data, to it.	91.50% (161)	8.50% (15)
2.	VR is a computer-generated simulation of a three-dimensional environment that can be interacted with in a seemingly real or physical way.	89.80% (158)	10.20% (18)

**Table 8:** Knowledge of examples of types of realities, in terms of percentage, %, and frequency, n. Correct answers are formatted in bold. VR: Virtual reality; AR: Augmented reality; MR: Mixed reality

Questions	Options	Frequency of Responses, n	Percentage of Responses, %
Which of the following scenarios is a good example of VR?	Microsoft HoloLens is a reality headset that overlays holographic images onto the real world	62	35.20%
	Oculus Rift offers an immersive gaming experience, enabling users to feel present in a computer	76	43.20%
	Pokemon GO is a game where virtual creatures appear in the real world through the use	38	21.60%
Which of the following scenarios is a good example of AR?	Microsoft HoloLens is a reality headset that overlays holographic images onto the real world	71	40.30%
	Oculus Rift offers an immersive gaming experience, enabling users to feel present in a computer	41	23.30%
	Pokemon GO is a game where virtual creatures appear in the real world through the use	64	36.40%
Which of the following scenarios is a good example of MR?	Microsoft HoloLens is a reality headset that overlays holographic images onto the real world	71	40.30%
	Oculus Rift offers an immersive gaming experience, enabling users to feel present in a computer	42	23.90%
	Pokemon GO is a game where virtual creatures appear in the real world through the use	63	35.80%

## Discussion

The objective of this study was to determine the level of knowledge that Pakistani HCPs have about AI and types of realities, as well as assess their awareness of and practice with AI. With rapid advancements being made in the field of AI, it is becoming increasingly common for this technology to be used to support the digital transformation of healthcare and provide evidence-based care [4]. On a global level, this shift towards integrating AI into healthcare makes it necessary to understand to what degree HCPs have knowledge about, awareness of, and practice with AI, since they are the means by which this technology is exposed to patients. At a national level in Pakistan, a developing country that is lagging behind in the implementation of AI into healthcare, understanding the relationship between medical consultants and AI can shed light on how to narrow the educational, research, and clinical gap of AI between Pakistan and the developed world [3].

About 69.9% of the participants in the study were male compared to 29% who were female, a ratio that corresponds with other studies. Others preferred not to disclose their gender [5,6]. The average age of participants was 42.0 years, similar to that of other studies [5-7]. 21% of the participants indicated that they have taken courses or training related to AI, a percentage that is slightly higher than in studies conducted in other developing countries such as Iran (2.2%) and India (6.5%), but still not a very high value, suggesting that there is a sufficient need for AI-related education within Pakistan’s medical landscape [8,9].

Approximately 97.7% of participants indicated that they own a smartphone or tablet, with 95.5% of participants indicating that they use the Internet daily, suggesting that Internet access is very prevalent amongst medical consultants. Other studies of similar nature did not measure this metric, likely assuming that most medical professionals had access to this technology. The majority (52.8%) of participants indicated that they both liked and felt confident using or working with computers and other devices, suggesting that there is a basic affinity for and efficacy with such technology. Other studies did not inquire about levels of basic technological competence, opting to jump straight into questions about AI, perhaps because it is more pertinent to the objective. Understanding technical affinity is important, however, in a nation such as Pakistan, where the same assumptions about this metric could not be made as they can be in developed countries. It is evident that medical consultants have a decently strong technical affinity, information that informs the overall discussion about AI in the country [1-7].

The majority of participants indicated that they either agree or strongly agree with the notion that AI will be of benefit to patients (75.6%) and had an overall positive or a very positive opinion about the use of AI in medicine (62%). This sentiment is shared across other studies, suggesting that Pakistan is similar to other nations in terms of attitude about AI in healthcare [6,9-11]. A common concern about AI is its potential to replace jobs. 67% of participants, however, indicated that they disagree with the notion that physicians will play a less important role in the therapy of patients in the future, a sentiment shared by participants in other studies [9,11-15]. This shows that medical consultants feel a certain degree of comfort that their job is something that AI will not be able to replicate. At the same time, 78.4% of participants agree or strongly agree with the notion that physicians know too little about AI to use it on patients, suggesting that although there is a favorable opinion about it, there should be more professional knowledge surrounding it prior to clinical implementation. It is no surprise, then, that 67.6% of participants are more afraid of a technical malfunction of AI than of a wrong decision by a physician, suggesting that although there is a favorable opinion about AI, it does not decrease the necessity of or trust in a physician.

**Figure 5:**
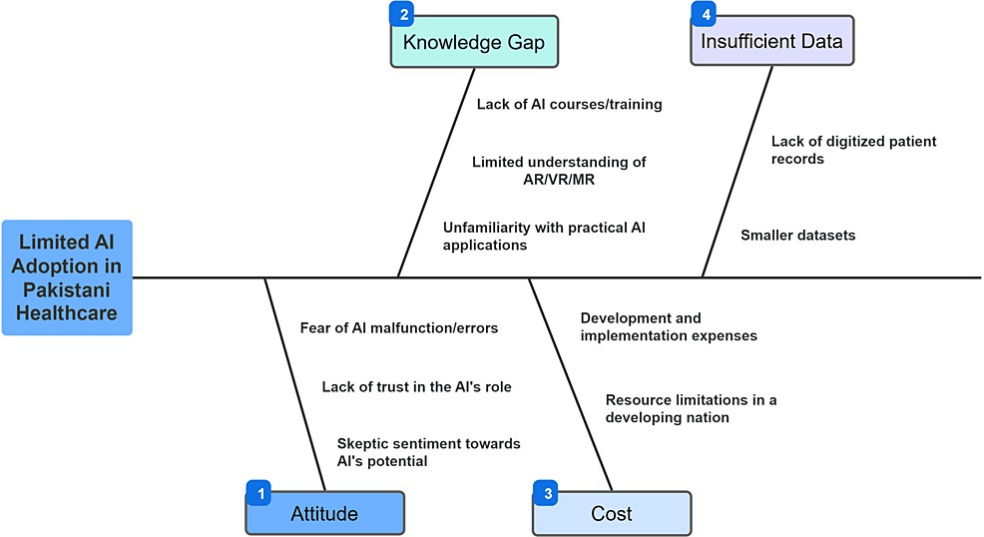
Fishbone model of causes of limited adoption of artificial adoption among Pakistani HCPs. Conception and creation by authors. HCP: Healthcare professional

Although the results also indicate that medical consultants in Pakistan have a solid understanding of what VR and AR are, the results clearly highlight that they lag in knowing which situation corresponds to which type of reality. This is an important finding because VR and AR have significant applications in medicine, much like AI, so having HCPs be familiar with such technologies is a prerequisite for implementing such technologies in Pakistan as well [2]. Other studies on AI did not include any questions about VR or AR, likely because such topics were outside the scope of studies focused primarily on AI alone. Authors propose a fishbone model of causes of low adoption of AI among HCPs of Pakistan (Figure 5).

Strengths and limitations

This study focused on medical consultants as opposed to medical students or medical professionals who are not at the consultant level. This ensured that the population being researched was composed of individuals who were knowledgeable about and established in their field, resulting in a more accurate understanding of the knowledge, attitudes, and practice of AI within the medical landscape of Pakistan. It had a decent male to female proportion, something that is valuable in studies that are conducted in male-dominated fields and countries like Pakistan. Although the sample of participants in the studies are being generalized to medical consultants across Pakistan, it is important to note that 94.9% of the participants were from the province of Punjab, suggesting that the vast majority of responses came from a single province and that generalizability should be tempered by that fact.

Clinical implications

It is derived that we must enhance educational initiatives to improve the literacy of AI and types of realities among HCPs, broadening awareness of AI applications beyond traditional domains. We must involve expert physicians in the development and validation of types of realities and AI systems to facilitate the effective and responsible implementation in our healthcare settings. To foster a coherent and scientifically grounded understanding of AI in healthcare, it is imperative to prioritize formal training courses aimed at medical schools and hospitals. These courses should be meticulously designed to ensure the effective dissemination of knowledge and skills related to AI applications in medicine. Targeted training initiatives should be implemented to facilitate hands-on experience with AI tools and methodologies, enabling doctors to confidently incorporate AI into their clinical decision-making processes. Furthermore, emphasis should be placed on instilling a strong ethical framework within medical education, ensuring that future physicians understand the ethical implications of AI use in healthcare and are equipped to uphold patient rights and professional values. This includes considerations such as privacy, data security, bias mitigation, and transparency in AI algorithms and decision-making processes. We must be effectively prepared for the transformative impact of AI and ensure that medical professionals are equipped to leverage these technologies for the benefit of patients and society. Future research should explore the impact of organizational readiness on technological adoption and educational strategies in health care.

## Conclusions

The results indicate that medical consultants in Pakistan have a familiarity with the concept of AI and its application to healthcare, as well as a general positive opinion about the technology. However, most are not comfortable with integrating this technology into healthcare at the moment due to concerns about reliability, safety, lack of testing and evidence-based practical use. They still believe that there is great potential for AI as a tool that works in harmony with a physician as opposed to in the place of a physician. Pakistan is on its way to the path towards incorporating AI into its medical infrastructure to the extent that the developed nations have. It has the potential to move past the initial stages of AI implementation and into more advanced modes of adopting AI. 
